# Exploring patients' and clinicians' experiences of video consultations in primary care: a systematic scoping review

**DOI:** 10.3399/bjgpopen20X101020

**Published:** 2020-03-18

**Authors:** Arun Thiyagarajan, Calum Grant, Frances Griffiths, Helen Atherton

**Affiliations:** 1 Honorary Research Fellow, Warwick Medical School, University of Warwick, Warwick, UK; 2 Medical Student, Warwick Medical School, University of Warwick, Warwick, UK; 3 Centre for Health Policy, University of the Witwatersrand, Johannesburg, South Africa; 4 Professor of Medicine in Society, Warwick Medical School, University of Warwick, Warwick, UK; 5 Associate Professor, Warwick Medical School, University of Warwick, Warwick, UK

**Keywords:** remote consultation, primary health care, telemedicine, patient satisfaction, physician—patient relations

## Abstract

**Background:**

Video consultation (VC) is an emerging consultation mode in general practice. The challenges and benefits of implementing it are not necessarily realised until it is in use, and being experienced by patients and clinicians. To date, there has been no review of the evidence about how patients and clinicians experience VC in general practice.

**Aim:**

The study aimed to explore both patients' and clinicians' experiences of VCs in primary care.

**Design & setting:**

A systematic scoping review was carried out of empirical studies.

**Method:**

All major databases were searched for empirical studies of any design, published from 1 January 2010 to 11 October 2018 in the English language. Studies were included where synchronous VCs occurred between a patient and a clinician in a primary care setting. Outcomes of interest related to experience of use. The quality of included studies were assessed. Findings were analysed using narrative synthesis.

**Results:**

Seven studies were included in the review. Patients reported being satisfied with VC, describing reduced waiting times and travel costs as a benefit. For patients and clinicians, VC was not deemed appropriate for all presentations and all situations, and a face-to-face consultation was seen as preferable where this was possible.

**Conclusion:**

The findings of this scoping review show that primary care patients and clinicians report both positive and negative experiences when using VCs, and these experiences are, to a certain extent, context dependent. VC is potentially more convenient for patients, but is not considered superior to a face-to-face consultation. Accounts of experience are useful in the planning and implementation of any VC service.

## How this fits in

There is increasing use of video consultation (VC) in primary care as an alternative to a face-to-face consultation and it potentially changes the experience of having a consultation for patient and clinician. A small number of studies where experiences of using VC were reported were found, and they showed that while it was convenient, it was not deemed appropriate in every situation. More nuanced findings may be achieved as use of video consulting spreads and more research emerges.

## Introduction

Internationally, video is used for conducting consultations in the routine delivery of health care, with use reported across a range of settings and clinical specialties, including specialist diabetes care,^1^ paediatric acute care,^2^ specialist palliative care services,^3^ primary care,^4^ clinical oncology,^5^ and mental health settings.^6^ Despite offering advantages relating to its remoteness and convenience for patients,^7,8^ adoption and uptake have been slow in healthcare settings.^9,10^ This has particularly been the case for primary care settings, where levels of use are low.^11,12^

At an international level, policymakers and the professional bodies representing primary care are encouraging the adoption of video as a routine way to consult with patients.^13–17^ This has been driven by the perceived benefits of using video for consulting with patients in a primary care setting; for example, improved access and convenience for patients^18^ in a setting where accessing a clinician can be challenging,^19^ the modernisation of primary care practice,^20^ and potential time-savings for clinicians who are facing increasing levels of demand for consultations.^21,22^ However, there have been concerns expressed by clinicians about VCs; for example, the potential for inequitable access to health care where patients cannot access and use the internet easily,^23^ that it challenges the role of clinicians,^24^ that there is a lack of clarity about what types of problem it would work best for, and the potential for technological and logistical problems.^20,25^

While there is evidence that patients and clinicians consider VC as a potentially acceptable method of consultation with a primary care clinician, much of this is based on hypothetical opinion rather than experience.^26–29^ Overall, it is not currently clear whether the perceived benefits and concerns are realised in practice for primary care settings. Research conducted in secondary care settings has shown that implementation of VCs into real-world settings is complex and context dependent.^10^ These complex and context dependent factors emerge as VC is used.

With experience a crucial element of how VC might be likely to work in practice and an indicator of what this use might look like, the current evidence was summarised on how VC is experienced by patients and clinicians in primary care, using a scoping review. To date, there has been no published review examining the reported experiences of those using VC in general practice.

## Method

A scoping review was conducted, which appled guidance from the Joanna Briggs Institute for Evidence Based Healthcare in the conduct of this review type.^30^ ^31^

### Research question

The research question was as follows: ‘what are the experiences of clinicians and patients when conducting VCs in primary healthcare settings?’

#### Inclusion criteria

The intervention of interest was the use of synchronous VC for a two-way communication. The comparator of interest was a usual method of consultation (face-to-face, telephone, email, any other two-way mode of consultation) or no comparator. Participants were all patients and/or carers and all staff having experienced two-way synchronous VCs in a primary care setting.

Included studies were published from 1 January 2010 to 11 October 2018, in the English language and from any geographical location. All types of empirical research study were included.

A cut off of 2010 was chosen as previous systematic reviews on VC have demonstrated low levels of published evidence before this date.^32^ A conceptual review and empirical research by one of the co-authors supports the slow growth of the evidence base in this field (although increasing) and demonstrates the low levels of usage of VC in primary care to date.^11,33^

#### Exclusion criteria

Studies were excluded where the intervention involved two-way synchronous VC between clinicians, asynchronous VC, or use of VC as a method of treatment. The study did not include the grey literature, unpublished research or published commentary, or discussion articles.

### Outcomes of interest

The outcomes of interest for this study were patient experience (for example, satisfaction, preference) and clinician experience (for example, satisfaction, attitudes, and service utilisation). The findings were used to map key concepts underpinning this research area to develop the conceptual boundaries of this topic.

### Search strategy

The following databases were searched: MEDLINE, Cochrane Central Register of Controlled Trials, specialised register of the Cochrane Effective Practice and Organisation of Care Group (EPOC), PubMed, EMBASE and CINAHL, and Web of Science.

The keywords and subject headings related to VCs in primary care. See Supplementary Appendix S1 for a copy of the Medline search strategy.

### Study selection

The titles and abstracts of all retrieved articles were screened by two reviewers independently. Then, the full text of relevant studies were examined by two reviewers independently. Where a difference in selection occurred, disagreements were resolved through discussion or with input from the third reviewer and the included studies were finalised.

### Data extraction

A standardised data extraction form was created for the data extraction process. Data were extracted on study design, population, the intervention, comparator, and the outcomes of interest.

### Quality assessment

The methodological quality of the studies were assessed independently by two reviewers using the Mixed Methods Appraisal Tool (MMAT). This tool is designed for systematic reviews that include qualitative, quantitative, and mixed-method studies and, therefore, allowed the application of one tool for all the studies in the review.^34^ Disagreement regarding study quality was resolved by discussion. The results of the tool were used to provide context on the included studies and the number of MMAT domains satisfied in each study are presented.

### Analysis

A narrative synthesis of the findings was conducted in the included studies. This offered a way to summarise together the findings of both quantitative and qualitative studies. The study followed the guidance of the Joanna Briggs^30^ Institute in structuring the narrative synthesis, developing categories, and adopting a textual approach to detailing the main findings.^35^

## Results

### Search results

**Figure 1.**
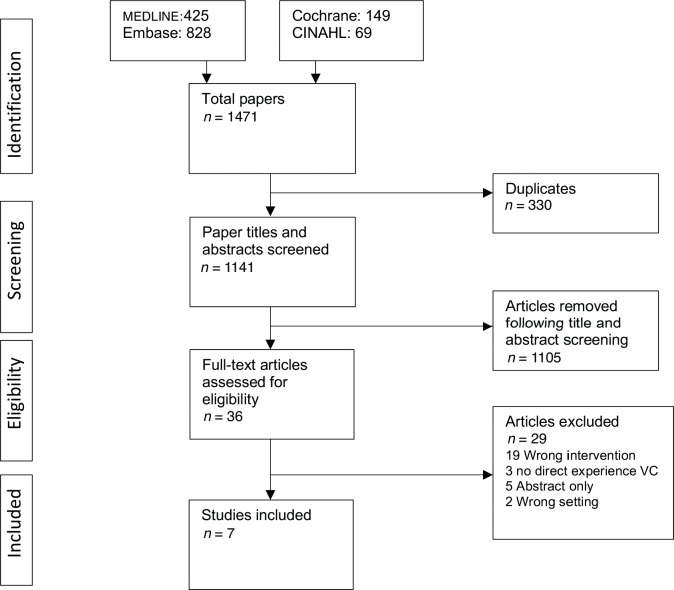
PRISMA diagram: papers included in the review. VC = video consultation.

A total of seven studies were included in the review. Figure 1 outlines the search process and how this final number was reached.

### Quality assessment

The overall quality of the studies included in this review was high (see Table 1). All studies had clear sources and processes of data, and had designs that were relevant to address the research question.

**Table 1.** Characteristics of included studies

**Author, year**	**Country study conducted**	**Study design**	**Participants**	**Quality assessment** **(criteria met**)
**Patients**	**Primary care staff**
Atherton *et al*, 2018^20^	UK	Ethnographic, case study	39	45	High 5/5
Brant *et al*, 2016^11^	UK	Cross-sectional, mixed methods	N/A	319	High 5/5
Glaser *et al*, 2010^36^	US	Cross sectional Survey	737	N/A	High 4/5 — derivation of findings from data not ideal due to length survey, subsequently final survey omits several questions
Hatton *et al*, 2018^37^	US	Cross-sectional survey	57 (26 VC, 31F2F)	N/A	High 4/5 — limited generalisability of patient population.
Polinski *et al*, 2016^38^	US	Cross-sectional survey	1734	N/A	High 5/5
Powell *et al*, 2017^39^	US	Qualitative interviews	19	N/A	High 5/5
Stahl and Dixon 2010^40^	US	Randomised control trial	175	4	High 4/5 – no blinding carried out

F2F = face to face. N/A = not applicable. VC = video consultation.

Where studies did not meet the domains of the MMAT, this was because some of the studies (2/3) that conducted surveys did not collect data around non-responders, including the reason for refusal of participation.^37,38^ One survey did not collect any data on the total number of patients that were invited to complete the survey.^38^

Five studies used VC as a study intervention.^36–40^ In all of these, VC was conducted via a conferencing platform on a smartphone, webcam, or tablet, synchronously between a patient and a clinician. Of these five, one study was a randomised control trial.^40^ Three studies were quantitative descriptive studies^36–38^ and one was a qualitative interview study.^39^ Two studies^11,20^ asked patients and/or clinicians about their perceptions of their use of VCs in a primary care setting where this was already available and both were qualitative study designs.^20,23^ See Table 1 for study details.

### Patients and VC

Four studies included in this reveiw looked at patient outcomes. Further results are available in Supplementary Table S1.

#### VC offering convenience and access

Convenience and improved access were identified as patient perceived benefits of VCs.^20,38,39^ Three studies, all set in the US, showed that patients had chosen VCs in certain circumstances; that is, to reduce travel costs or to minimise time waiting for an appointment or for certain types of condition.^38–40^

#### Satisfaction with VC as a medium for consultation

In a cross-sectional survey, approximately 94–99% of patients were reported to be ‘very satisfied’ after a VC, with 95% (521/551) of patients stating that they would definitely personally use VCs again.^38^ A survey of patients who had a consultation with a pharmacist compared satisfaction between those patients seen face-to-face and those seen via VC. There was no statistically significant difference between the two groups in relation to how patient-centred the communication was, and the patient perception of the clinician's competence and skill.^37^ In an interview study, patients reported that they felt able to establish a rapport with their clinician.^39^

A randomised control trial comparing video with routine visits reported that patients were more satisfied overall with face-to-face consultations when assessed using a Likert scale (1 = poor, 5 = excellent)(4.6–4.3, *P*<0.0001) and that patients with chronic conditions were more likely to prefer a face-to-face consultation, this was reported by the authors as a significant *P* value for the difference between groups (**P** = 0.01).^40^ This matched the findings from a UK qualitative study, which found that patients regarded a face-to-face consultation as the ‘gold standard’.^20^ Patients reported difficulties in finding private places to conduct a VC, and this potentially affected the ability to share sensitive information.^39^ Technological issues, such as time lag in images and audio, connections and password logins, hindered fluidity in consultation for some patients.^39^

#### Patient characteristics and VC

Two studies explored patient characteristics. In the randomised controlled trial (RCT) by Stahl and Dixon,^40^ age and sex of the patient did not have an effect on satisfaction levels, but in the study by Polinski *et al*,^38^ female sex was a predictor for preferring a VC. In the interview study by Powell *et al*, older patients reported that VC allowed them to avoid the burden of travelling to consultations.^39^

### Clinicians and VC

Four of the included studies looked at clinician outcomes. Further results are available in Supplementary Table S1.

#### Clinician satisfaction with VC for delivering care

Glaser *et al* ^36^ found that 88.2% (650/737) of participating clinicians felt that a VC visit had improved the patients prognosis, and that 89.4% of clinicians (652/729) agreed clinical decision making was successfully accomplished using VC. In the RCT by Stahl and Dixon clinicians felt that their ability to take a history was not impaired.^40^ However, the same study found that clinician satisfaction with VC was reduced when new treatments were initiated, and this was measured using a Likert scale (1 = poor, 5 = excellent) (4.2 versus 4.5; *P* = 0.02) and that clinicians felt less satisfied with their ability to order appropriate laboratory tests when consulting via VCs.^40^

In two studies, one RCT^40^ and an interview study,^20^ clinicians reported a preference for face-to-face consultations, which matched findings observed in patients. Reasons given for this included poor physical exam capabilities, reduced ability to choose correct investigations, and challenges using this medium for assessing mental health patients.^40^ Clinicians made individual clinical judgements based on the characteristics of patients and the condition they presented with.^20^

In some cases, for example in rural locations, the geographical need for a remote consultation meant clinical suitability was not a factor in deciding whether to use VC.^20^

#### Clinicians’ perspectives of patient satisfaction

In two studies,^36,40^ clinicians rated their experience with VCs highly with one finding that 83.6% (616/737) of clinicians believed that patients were completely or generally satisfied and 88.5% (652/737) agreeing or completely agreeing that successful clinical decision-making was achieved through VCs.^36^ Clinicians expressed concerns that certain groups, such as those who are disadvantaged and vulnerable, may struggle to engage in VC, which would lead to an unintentional inequality in healthcare delivery.^11,20^

## Discussion

### Summary

The available evidence demonstrates that patients and clinicians are largely satisfied with VC, although this is dependent on the nature and circumstance of the consultation. Patients felt that VCs are patient-centred and that it is possible to build rapport; however, for both patients and clinicians, the face-to-face consultation is still preferred. Convenience and access are the key benefits for patients, but not all types of patients are engaging in VC and these benefits may not be afforded to all patients.

### Strengths and limitations

This scoping review is timely in the current policy context and has mapped the emerging evidence on the patient and clinician experience of VC. The low number of existing studies highlights the paucity of evidence in this area and limits the extent of the findings.

While a systematic search was conducted, the review was limited to studies from 2010 onwards. Expanding the inclusion criteria to include studies prior to 2010 may have resulted in studies that contained relevant data. The study examined the experience of staff delivering the consultations, but did not include other staff, such as practice staff members who may be involved in set up and scheduling of VC, and this limits the applicability of the findings to the wider general practice setting. The participants in the included studies may be viewed as self-selecting populations, and this may introduce a confirmation bias with regards to the baseline attitude to VC.

### Comparison with existing literature

It was identified that patients felt they were able to establish a rapport with their clinician and this also has been demonstrated in studies outside of primary care.^41^ In a study of VC versus standard home care in families with premature infants, patients reported positive experiences; however, for clinicians, views were mixed and there were challenges in encouraging nurses to accept the use of VC.^42^ In this review, a similar pattern was seen whereby patients were largely satisfied, but for clinicians their experience was more varied and dependent on several factors.

In 2013 a vignette study using mocked-up patient scenarios found that one-third of participating GPs were supportive of VC, one-third ambivalent, and one-third against the use of VC in primary care. GPs who worked in larger, more rural practices were more inclined to support its use.^4^ These findings correlate with the findings of this review, where mixed opinions were observed from clinicians on the use of VC and these opinions were often context dependent.^20,39,40^ The experience of patients and clinicians was shown to be context dependent in primary care settings; for example, dependent on the condition, the patient type, and on the individual and their personal perspective. This fits with the findings of research conducted in other healthcare settings, where real-world evaluations have identified this variability and its influence on how VC is ultimately used in practice.^10^

### Implications for research and practice

Patient views vary with regard to how they would preferentially use VC, with some suggesting it would be their ‘go-to’ visit and others preferring VC as a supplementary way to consult with their primary care doctor.^39^ The review suggests VC has a role for certain types of consultation and can in some cases be more convenient for patients. Acknowledging this variation is key when planning a VC service.

Future research should use patients' and clinicians'experiences as a way to best design a VC service, allowing for variation according to contextual factors such as population mix and patient condition.

The findings of this scoping review show that primary care patients and clinicians report both positive and negative experiences when using VCs and these experiences are to a certain extent, context dependent. VC is potentially more convenient for patients, but is not considered superior to a face-to-face consultation. There are key factors that service providers should consider when setting up synchronous VCs within their service, making consideration for the type of condition and the needs of their patient population.

## Trial registration number

The review protocol is registered with PROSPERO (CRD42018100032).
